# Efficacy of Supervised Exercise Therapy for Intermittent Claudication in a Case With Buerger’s Disease

**DOI:** 10.7759/cureus.43537

**Published:** 2023-08-15

**Authors:** Daisuke Komiya, Kohji Iwai, Tomokazu Ohno

**Affiliations:** 1 Division of Rehabilitation, Omuta City Hospital, Fukuoka, JPN; 2 Division of Physical Therapy, Faculty of Care and Rehabilitation, Seijoh University, Aichi, JPN; 3 Division of Vascular Surgery, Omuta City Hospital, Fukuoka, JPN

**Keywords:** supervised exercise therapy, intermittent claudication, peripheral arterial disease, thromboangiitis obliterans, buerger’s disease

## Abstract

Herein, we report a case of intermittent claudication (IC) caused by Buerger's disease (thromboangiitis obliterans {TAO}), which we treated using supervised exercise therapy (SET). The patient was a 58-year-old male with a history of smoking who presented with IC and resting pain in the right lower extremity, which had led to necrosis of the right first toe eight years prior to presentation. The non-healing right first toe was amputated and the patient underwent angiogenesis therapy in the right lower extremity. Despite continued strict smoking cessation and antiplatelet medication, the patient presented with IC of the left lower extremity eight years after the previous symptoms. Therefore, the patient underwent SET once a week (40 min per session) for five months, resulting in a total of 21 sessions. Consequently, the patient’s walking ability and quality of life (QoL) significantly improved. These results suggest that SET is an effective treatment for TAO-induced IC. However, further studies are required to demonstrate its efficacy.

## Introduction

Buerger’s disease (thromboangiitis obliterans {TAO}) is a form of segmental inflammatory thrombosis that primarily affects medium-to-small-sized vessels at the distal ends of arteries and veins [1]. This disease is prevalent among young male smokers [2] and tends to progress rapidly, often leading to peripheral necrosis [2]. Although the exact cause of TAO remains unknown, smoking has been strongly linked to its onset and progression [3]. In Japan, TAO was designated as an intractable disease in 1975 [1]. Notably, the number of TAO patients in Japan has decreased since the early 2000s, and the total number of cases was 7,043 in 2014 [1]. Furthermore, the prevalence rate per 100,000 population was 5.54% in Japan, while the prevalence rate in all patients with peripheral vascular occlusive disease was estimated at 6.12% [1]. The management of TAO is centered around strict smoking cessation practices, which involve avoiding passive smoking, and pharmacological interventions such as the use of antiplatelet agents [4].

The diagnostic criteria for TAO in Japan include intermittent claudication (IC). IC diminishes the physical activity, walking distance, and quality of life (QoL) of patients, which affects their social, emotional, and mental health [5]. Therefore, IC is a clinically significant symptom of TAO, and the reason supervised exercise therapy (SET) is recommended as an initial treatment [4]. However, to our knowledge, no previous studies have investigated the impact of SET on IC caused by TAO. Additionally, studies on IC caused by peripheral arterial disease (PAD) have generally not explicitly included TAO cases. As mentioned above, some points remain unclear regarding the efficacy of SET. Therefore, we report a clinically significant case in which SET was effective in treating IC caused by TAO.

## Case presentation

Clinical course

**Figure 1:**
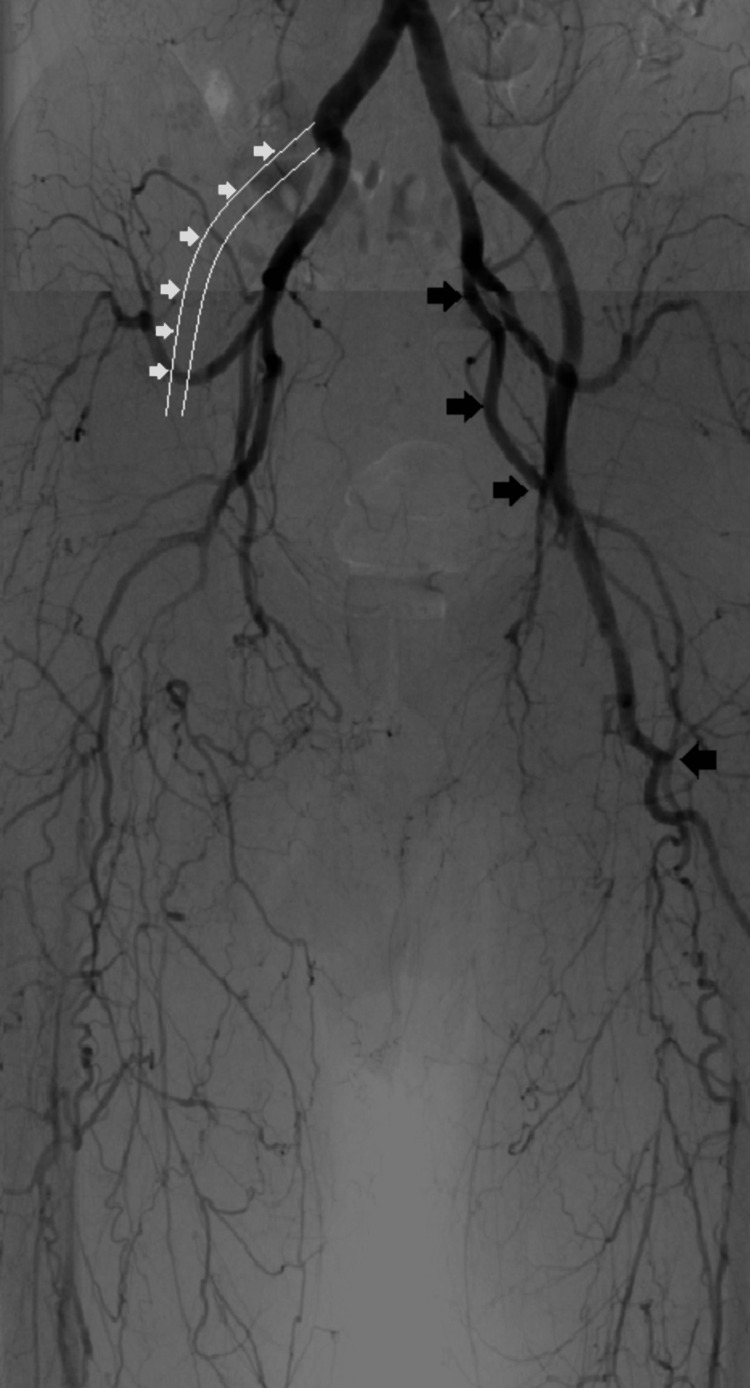
Angiographic image obtained at the time of TAO diagnosis The right external iliac artery shows a total occlusion at its origin (white arrows). The left common femoral artery is also occluded. Additionally, typical collateral circulation of thromboangiitis obliterans (TAO) originating from the left internal iliac artery is observed (black arrows).

The patient was a 58-year-old male with a 45-year history of smoking 30 cigarettes/day, an occupation of long-distance truck driving, and a body mass index of 22.1. The presentation symptoms included IC and resting pain in the right lower extremity, which had led to necrosis of the right first toe eight years prior to presentation. The patient had several comorbidities, including hypertension, chronic kidney disease, and chronic sinusitis, and angiography revealed complete occlusion of the right external iliac artery at its origin. In addition, the left common femoral artery was occluded and typical collateral circulation of TAO originating from the left internal iliac artery was observed (Figure 1).

The patient was diagnosed with TAO and underwent angiogenesis therapy for the right lower extremity and a nonhealing right first toe amputation. Strict instructions were issued recommending smoking cessation, which improved the symptoms. Subsequently, sarpogrelate hydrochloride (300 mg/day) and cilostazol (200 mg/day) were administered and the patient has been monitored since then.

**Figure 2:**
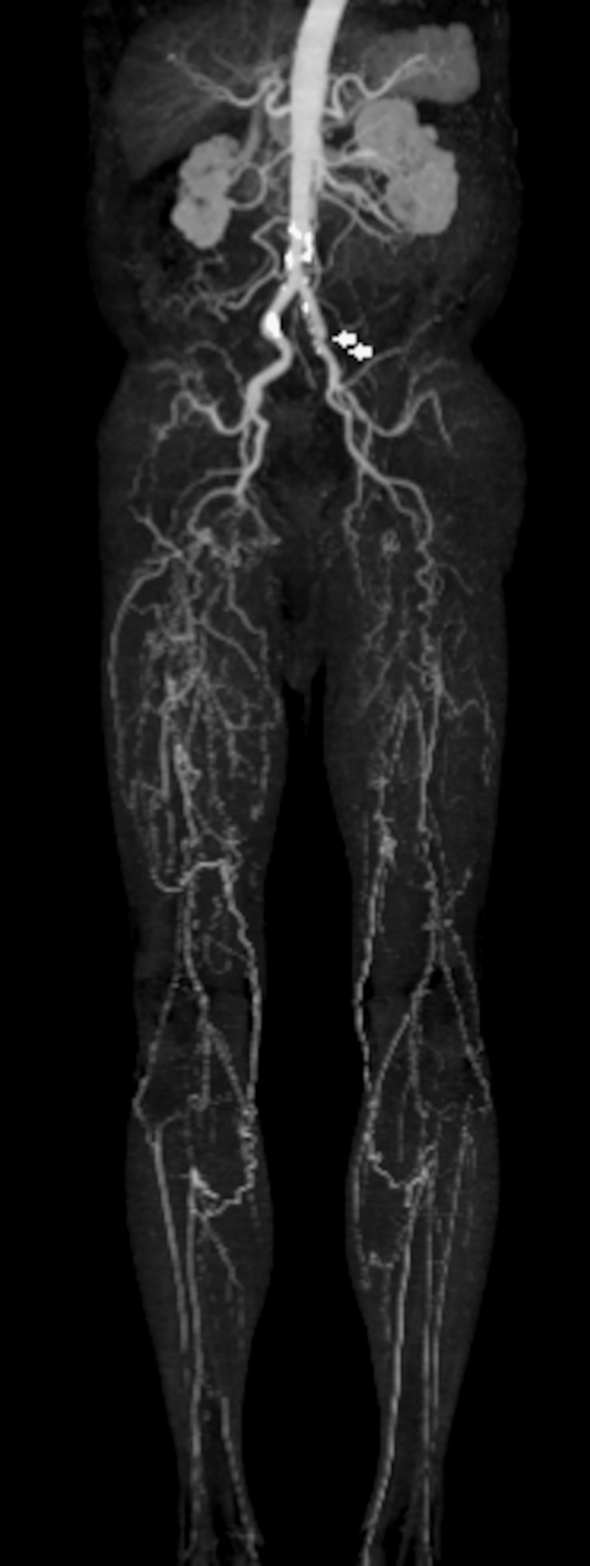
Contrast-enhanced computed tomography image The image is showing exacerbation of IC in the left lower extremity. The left external iliac artery, which was patent at the initial visit, is now occluded at its origin (white arrows).

The patient revisited the hospital due to IC exacerbation caused by pain in the left leg. The ankle-brachial index (ABI) could not be measured. It was found that the left external iliac artery, which was patent at the initial visit, was occluded at its origin. Therefore, the left lower extremity was perfused only with collateral circulation from the left internal iliac artery (Figure 2).

To quantitatively evaluate the patient's walking ability and walking impairment, we measured the initial claudication distance (ICD) and absolute claudication distance (ACD) using the progressive load treadmill test by Gardner et al., which has already been reported to be valid in stable peripheral vascular occlusive disease [6]. Additionally, we calculated the Walking Impairment Questionnaire (WIQ) score. The WIQ is a disease-specific questionnaire that evaluates walking impairments in patients with IC caused by PAD [7]. A higher WIQ score indicates a better condition. Importantly, the WIQ has been reported to be an effective tool for detecting changes in the daily walking ability of patients with IC, indicating improvement or decline [8]. The Japanese version of the WIQ has also been reported to be an effective tool for evaluating treatment responses in patients with PAD [9]. The results of the initial evaluation were as follows: the walking ability was 220 m for the ICD and 470 m for the ACD; the WIQ scores were 50, 19, 22, and 67 for pain, walking distance, walking speed, and climbing stairs, respectively, resulting in a total score of 158. Furthermore, we utilized the Vascular Quality of Life Questionnaire (vascuQoL)(A3), which has already been reported as a valid tool for quantitatively evaluating the QoL in patients with chronic lower limb ischemia [10]. In addition, the vascuQoL is a disease-specific QoL assessment scale consisting of 25 questions [10]. In this case, the average score of each domain was calculated, and the higher the score, the higher the QoL. The average vascuQoL score for each domain in the patient was 2.9, with a total score of 70. This case report was conducted in accordance with the principles of the Declaration of Helsinki, with strict adherence to the ethical considerations and standards for medical research involving human subjects. The patient provided informed consent to participate in the study.

Supervised exercise therapy

To determine the presence of coronary risk factors, we initially reviewed the patient's medical history. We identified two coronary artery disease risk factors: smoking and hypertension. Therefore, we asked if the patient experienced chest pain at rest or during exertion and confirmed that this was not the case. The exercise protocol involved the use of a treadmill with the speed and slope adjusted to induce moderate leg pain within 10 minutes. Once moderate pain appeared, the exercise was stopped and the patient was instructed to rest until the pain disappeared. When the patient was able to walk for 10 minutes or longer under these conditions, the speed and slope were gradually increased. SET was performed once a week, and each session lasted 40 minutes, including breaks. Blood pressure, pulse rate, and percutaneous arterial oxygen saturation were measured before and after SET, and chest symptoms were recorded during SET. In total, SET was conducted 21 times over five months. The patient was also advised to perform home exercises by walking for at least 30 minutes, three times a week. However, the execution of these home-based exercises was not monitored. All treatments and instructions were provided by the same physiotherapist.

Follow-up results

**Figure 3:**
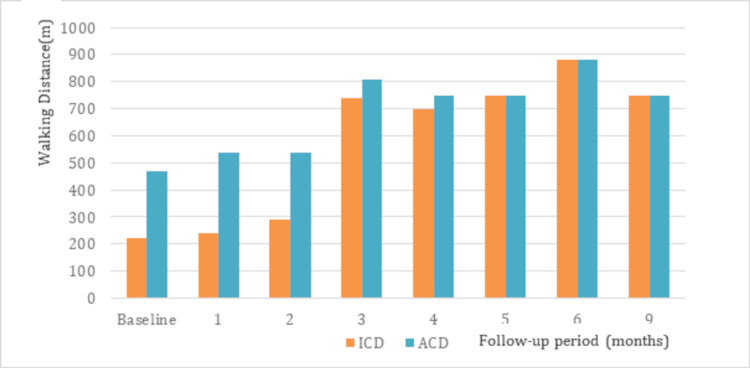
Changes in walking distance over time ICD, initial claudication distance; ACD, absolute claudication distance

**Figure 4:**
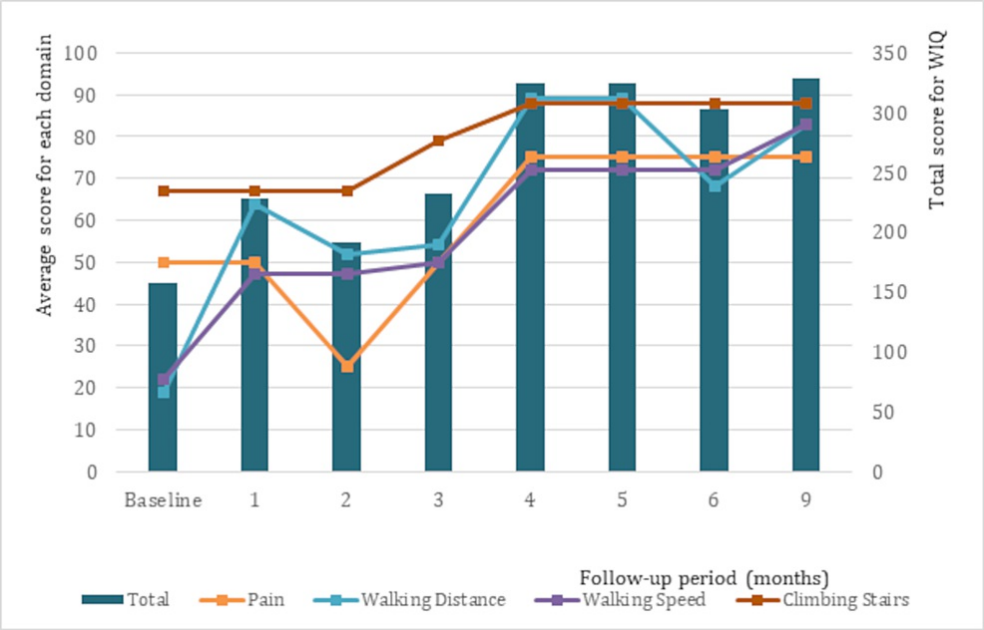
Changes in the WIQ score over time The left axis indicates the score for each domain and the right axis shows the total score. WIQ, walking impairment questionnaire

**Figure 5:**
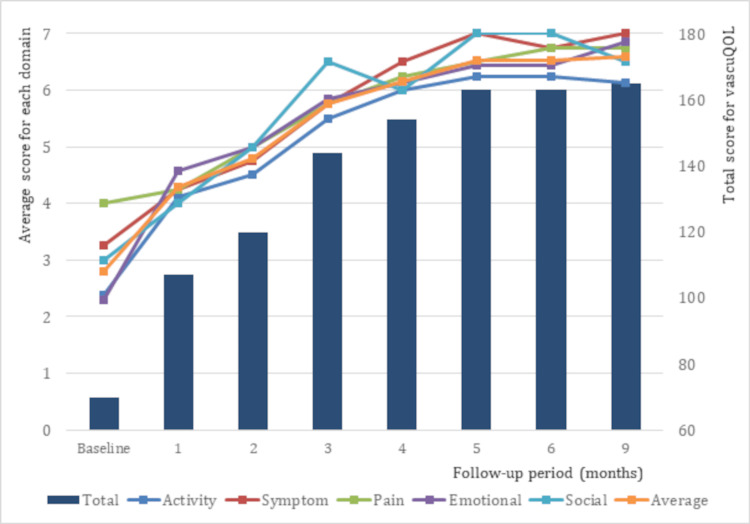
Changes in the vascuQoL score over time The left axis indicates the score for each domain and the right axis shows the total score.

The evaluation conducted at the five-month follow-up showed an ICD of 750 m and an ACD of 750 m. The WIQ scores for pain, walking distance, walking speed, and stair climbing were 75, 89, 72, and 88, respectively, with a total score of 324. Additionally, the vascuQoL scores averaged 6.52 for each domain, with a total score of 163. At the six-month follow-up, the ABI for the right and left limbs were 0.69 and 0.63, respectively. Moreover, treadmill tests performed at the six- and nine-month follow-ups showed similar distances for the ICD and ACD, with values of 880 m and 750 m, respectively. Figure 3 shows the changes in ICD and ACD from the treatment period to follow-up, while Figures 4-5 show the changes in the WIQ and vascuQoL scores, respectively.

## Discussion

Herein, we presented a case demonstrating the efficacy of SET in treating IC caused by TAO. Despite strict smoking cessation and oral antiplatelet therapy, the patient experienced IC exacerbation eight years after diagnosis. The 2017 guidelines for the management of vasculitis syndrome recommend SET by a physiotherapist as a treatment for TAO [11]. However, as this cites treatment recommendations for arteriosclerosis obliterans (ASO) verbatim, it may not be directly applicable to patients with TAO. Additionally, the guidelines do not provide a specific scale to assess the therapeutic effect of SET on IC caused by TAO. As the efficacy of SET for IC caused by TAO remains unclear, the quantification of its efficacy with ICD, ACD, WIQ, and vascuQoL in this case report is clinically important.

In this case, the patient underwent SET for five months and experienced approximately 3.4- and 1.6-fold increases in ICD and ACD, respectively. The minimum important difference (MID) for ACD, determined for patients with PAD, was 305 m [12]. In this case, the observed increase in ACD after SET was clinically meaningful and indicated improved walking ability. Two mechanisms might have contributed to this improvement. The first involves the enhancement of vascular endothelial and mitochondrial functions, which are thought to increase blood flow to the leg muscles in the lower extremities and improve the aerobic capacity of the microvasculature [13,14]. The above mechanism may be involved in the improvement of the ankle-brachial index (ABI) to a measurable level after SET. Regarding the second mechanism, habitual exercise improves weakness in the plantar flexors caused by atrophy [15,16] and reduces the loss of propulsion during walking [17]. Furthermore, in this case, the vascuQoL score improved significantly, from 2.9 to 6.52 in each domain and from 70 to 163 for the total score. The MID for vascuQoL improvement in pain and activity areas was found to be 1.48-1.91 and 1.55-2.2, respectively [18]. Therefore, SET may have led to a reduction in pain and subjective symptoms in the patient, which ultimately contributed to an improvement in QoL.

Necrotic lesions in TAO typically do not develop or recur in patients aged >60 years [19]. However, in this case, recurrence occurred even after medication and smoking cessation. Therefore, regular and quantitative evaluations are required in these patients. Additionally, it is important to note that the WIQ and vascuQoL scores used in this report were originally developed to assess IC in patients with ASO [7,10], and most clinical studies on IC have focused on these patients. Therefore, in future studies, it is necessary to accumulate a larger number of cases and investigate the effect of SET on IC caused by TAO.

## Conclusions

Our case report documents a noteworthy case of TAO with IC recurrence in the contralateral leg, eight years after the initial diagnosis, despite the patient adhering to strict smoking cessation, antiplatelet drug administration, and angiogenic therapy. In this case, we employed SET, and our findings demonstrated a significant quantitative improvement in the patient's walking ability and QoL. Throughout the five-month follow-up period, a notable enhancement was observed in walking ability and QoL, which continued even at the nine-month follow-up. These results indicate that SET is an effective treatment for IC caused by TAO. This case holds clinical importance, underscoring the possibility of disease progression several years later, even in patients who maintain standard treatment and experience symptom remission. In such cases, SET represents a promising non-invasive treatment option for IC, offering ease of implementation. However, further studies involving a larger accumulation of cases are necessary to establish the full efficacy of SET.
